# Prediction of very late arrhythmia recurrence after radiofrequency catheter ablation of atrial fibrillation: The MB-LATER clinical score

**DOI:** 10.1038/srep40828

**Published:** 2017-01-20

**Authors:** Nebojša Mujović, Milan Marinković, Nebojša Marković, Alena Shantsila, Gregory Y. H. Lip, Tatjana S. Potpara

**Affiliations:** 1 Cardiology Clinic, Clinical Center of Serbia, Višegradska 26, Belgrade, Serbia; 2 School of Medicine, University of Belgrade, Dr Subotića 8, Belgrade, Serbia; 3 University of Birmingham Institute of Cardiovascular Science, City Hospital, Birmingham, United Kingdom

## Abstract

Reliable prediction of very late recurrence of atrial fibrillation (VLRAF) occuring >12 months after catheter ablation (CA) in apparently “cured” patients could optimize long-term follow-up and modify decision-making regarding the discontinuation of oral anticoagulant therapy. In a single-centre cohort of consecutive patients post radiofrequency AFCA, we retrospectively derived a novel score for VLRAF prediction. Of 133 consecutive post AFCA patients (mean age 56.9 ± 11.8 years, 63.9% male, 69.2% with paroxysmal AF) who were arrhythmia-free at 12 months (excluding 3-month “blanking period”), 20 patients expirienced a VLRAF during a 29.1 ± 10.1-month follow-up, with a 3-year cumulative VLRAF rate of 31.1%. The MB-LATER score (**M**ale, **B**undle brunch block, **L**eft atrium ≥47 mm, **T**ype of AF [paroxysmal, persistent or long-standing persistent], and **ER**-AF = early recurrent AF), had better predictive ability for VLRAF (AUC 0.782) than the APPLE, ALARMc, BASE-AF2, CHADS_2_, CHA_2_DS_2_VASc or HATCH score (AUC 0.716, 0.671, 0.648, 0.552, 0.519 and 0.583, respectively), resulted in an improved net reclassification index (NRI) of 48.6–95.1% and better identified patients with subsequent VLRAF using decision-curve analysis (DCA). The MB-LATER score provides a readily available VLRAF risk assessment, and performs better than other scores. Validation of the MB-LATER score in other cohorts is underway.

Catheter-ablation (CA) is superior to antiarrhythmic drugs (AADs) in patients with symptomatic atrial fibrillation (AF), but AF may re-occur despite multiple re-do procedures and recent technical improvements^1^,^2^. Most arrhythmia recurrences are identified within the first year after the procedure, while further long-term follow-up strategy for initially “successfully” treated patients is less well defined and inconsistently reported^1^,^2^,^3^,^4^,^5^,^6^,^7^,^8^,^9^.

Several studies described a progressive increase in the arrhythmia recurrence rates over time in patients with initial post-ablation suppression of AF^4^,^5^,^6^. It is reported that 10–40% of patients who were arrhythmia free within the first post-procedural year experienced very late recurrence of AF (VLRAF) over long-term follow-up^2^,^3^,^4^,^5^,^6^,^7^,^8^,^9^. Several scoring systems (i.e., the APPLE, ALARMc and BASE-AF2 score) were recently proposed to predict the short-term risk of AF recurrence after the procedure^10^,^11^,^12^. However, none of these scores have addressed VLRAF occurring in patients who were free of AF at 1 year post procedure. Importantly, reliable identification of increased risk of VLRAF could strongly influence decision-making regarding the long-term use of oral anticoagulation and/or AAD therapy as well as the long-term monitoring strategy after the procedure^1^,^2^. Due to altered risk/benefit ratio, multiple AF ablation procedures may not be justified in patients at high risk of recurrent AF, and reliable prediction of VLRAF could optimize the patient selection for re-do CA^1^,^2^,^9^,^11^.

Our objectives were to determine the incidence and time-course of VLRAF after AF ablation and to derive a simple clinical score for prediction of VLRAF in a cohort of consecutive AF patients with a regular, structured follow-up for more than 1 year post AF ablation.

## Methodology

### Patient selection

We retrospectively analysed 212 consecutive adult patients who underwent a total of 277 radiofrequency (RF) CA procedures for electrocardiographically (ECG) documented symptomatic non-valvular AF resistant to ≥1 Class IC or III AADs, between February 2012 and June 2016 in the Clinical Centre of Serbia. All methods were performed in accordance with relevant guidelines and regulations. All patients provided written informed consent and the study protocol was approved by the Clinical Centre of Serbia Ethics Committee.

Baseline data were obtained from patient history, medical records and the hospital electrophysiology (EP) electronic database. Paroxysmal AF (PAF) was defined as AF that spontaneously terminated within 7 days, persistent AF (PeAF) as AF that required cardioversion or lasted ≥7 days and longstanding persistent AF (LSPeAF) as AF lasting >1 year^1^. The term “non-paroxysmal” AF (NPAF) included both PeAF and LSPeAF. The time since first-diagnosed AF to ablation procedure was >6 months in all patients. Patients with PAF had at least 2 symptomatic AF episodes in the last 6 months before the procedure.

Post-procedural finding of any symptomatic or asymptomatic atrial tachyarrhythmia (AF, atrial tachycardia [AT] and/or typical atrial flutter [AFL]) lasting >30 sec was considered the arrhythmia recurrence^1^,^2^. The recurrences registered within the 3-month “blanking period” after ablation were classified as the early recurrence of AF (ERAF)^1^,^2^. The off-AADs recurrences occurring after the “blanking period” but between 3 and 12 months after the procedure were considered the late recurrence of AF (LRAF), and the recurrences documented more than 12 months after the procedure were classified as VLRAF^1^,^2^,^7^,^8^,^9^. Patients with LRAF or VLRAF were offered a redo CA procedure or re-institution of AAD therapy.

### The VLRAF study group

**Figure 1:**
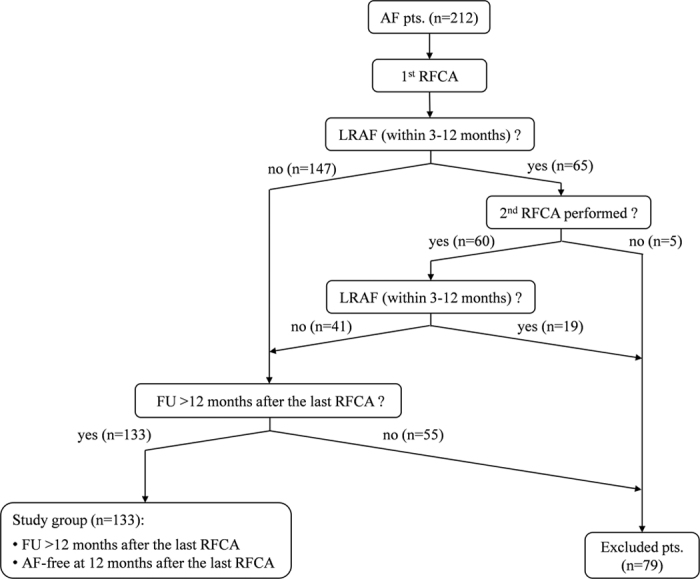
Study flow-chart. AF = atrial fibrillation; RFCA = radiofrequency catheter ablation; FU = follow-up; LRAF = late recurrence of AF (between 3 and 12 months after ablation); ERAF = early recurrence of AF (within the first 3-month “blanking period” after ablation).

The study flow-chart is presented in Fig. 1. On 1^st^ July 2016, all patients with a follow-up of >12 months after the last AF ablation were included in the present study, provided that they were free of LRAF since the last AF ablation (n = 103 patients post first ever AF ablation, n = 30 post a single re-do AF ablation).

### Preprocedural work-up

All patients received an oral anticoagulant for ≥6 weeks before the procedure^1^. All patients underwent pre-procedural transthoracic echocardiography, exercise stress testing, 24-hour Holter-monitoring and chest computed tomography (CT). The estimated glomerular filtration rate (eGFR) was calculated using the Modification of Diet in Renal Disease (MDRD) formula^10^. The left atrial (LA) dimension index was derived as the ratio of the anteroposterior LA diameter and body surface area (mm/m^2^)^13^. Metabolic syndrome was defined as presence of impaired fasting glucose (≥5.6 mmol/l) and/or type 2 diabetes mellitus plus two of the four following criteria: 1) body mass index ≥25 kg/m^2^, 2) hypertension, 3) serum triglycerides level ≥1.7 mmol/l and 4) HDL-cholesterol level <1.03 mmol/l for men or <1.29 mmol/l for women^14^. Definition of current cigarette smokers included current smokers and former smokers abstinent for less than 6 months^12^.

### Ablation procedure

All AADs were discontinued for ≥5 half-lives before the procedure, except for amiodarone which was stopped >1 month prior to ablation. The procedure was performed under deep sedation with midazolam, fentanyl and/or propofol. A quadripolar or decapolar catheter was inserted into the distal coronary sinus (CS), for stimulation and electroanatomical reference. Unfractionated heparin was administered intravenously after the transseptal puncture to maintain an activated clotting time between 280 and 350 seconds during the procedure. Navigation of the ablation catheter was performed with a long steerable sheath (8.5 Fr Agilis NXT, St Jude Medical, MN, USA), and pulmonary vein (PV) activity was assessed with a circular 10–20-polar catheter inserted through the long non-steerable sheath. After selective PV angiography, an anatomical LA map was created, and fusion with the CT scan was performed (NavX Ensite Classic and Velocity, St Jude Medical). All procedures were performed using RF energy and a 4-mm externally irrigated-tip catheter (CoolFlex or CoolPath Duo, St Jude Medical). The setup for LA ablation was: 43 °C and 30 W with flow rate 17 ml/min. All patients initially underwent circumferential antral PV isolation 1–2 cm outside from the ipsilateral PV ostia. RF applications on the anterior and posterior wall lasted 40–60 s and 20–40 s, respectively. In patients with large signals or spontaneous ectopic activity from superior vena cava (SVC), isolation of SVC was completed, too. In patients without AF termination after trigger isolation and in PAF patients with inducibility of AF lasting >10 min (inducibility was tested using repeated atrial burst pacing with cycle length of 200 ms for 5–10 sec), additional stepwise substrate ablation was performed. Substrate modification consisted of linear ablation (roof line and mitral isthmus line) followed by the LA complex fractionated atrial electrogram (CFAE) ablation and then the right atrial CFAE ablation. Endocardial LA linear ablation was performed with contiguous RF applications up to the linear block or maximum 30 minutes of RF delivery per line. Epicardial RF ablation within the CS was performed with a limit of 18–25 W. The CFAE were visually identified and ablated until their local elimination. If extensive ablation converted AF to organized AT, maping and ablation of AT was attempted. If this approach did not terminate arrhythmia, external cardioversion was performed, with revision of all lesion sets and additional touch-up ablations to close the conduction gaps. In patients with documented typical AFL, linear ablation of cavo-tricuspid isthmus (CTI) was performed. Linear ablation of CTI was performed with 35–40 W with the end-point of bidirectional conduction block. All patients were observed 20–30 min following the last RF application for final EP study.

### Follow-up

Oral anticoagulants and AAD that have been used before ablation were post-procedurally administered to all patients for 3 months following CA. After this “blanking period”, oral anticoagulation was continued in patients with a CHA_2_DS_2_-VASc score of ≥2, while AAD was discontinued in all patients.

In all patients, scheduled follow-up visits consisting of physical examination, 12-lead ECG and 24-hour Holter-recording were performed at discharge, 1, 3 and 6 months after the procedure, and every 6 months thereafter. Patients were instructed to obtain an ECG in case of symptoms suggestive of arrhythmia recurrence. In patients who experienced symptoms suggestive of arrhythmia recurrence, an additional non-invasive work-up was conducted, primarily by a supplementary 24–48-hour Holter-monitoring. If, in these patients, arrhythmia recurrence was not documented in this way, a more extensive rhythm monitoring was performed, including 7-day Holter-monitoring, event recorder use for 2–4 weeks, exercise stress testing and hospital admission for a 2–3-day observation.

### Statistical analysis

All continuous variables were presented as mean (±standard deviation) and categorical variables were summarized as percentages. Continuous variables with normal or asymmetrical distributions were compared using unpaired Student’s t-test or Mann-Whitney U test, respectively. Categorical variables were compared using Chi-square test or Fischer’s exact test, as appropriate. The association of the clinical variables listed in Table 1 with VLRAF occurrence during follow-up was analysed using univariate Cox’s proportional hazards regression model. The score for VLRAF risk assessment was subsequently derived from selected clinical variables which were significantly associated with the occurrence of VLRAF on univariate analysis. Continuous variables within the score (that is, the anteroposterior LA diameter in millimetres) were dichotomized at optimal cut-off value using the receiving operator characteristic (ROC) curve analysis. The area under the curve (AUC) was used as an indicator of predictive value of the tested scores. Decision curve analysis was used to assess clinical usefulness^15^. Integrated Discrimination Improvement (IDI) and Net Reclassification Improvement (NRI) were used to test improvement in predictive accuracy of the MB-LATER score^16^. The VLRAF-free survival curves for patient subgroups with different MB-LATER score values were estimated by the Kaplan-Meier method and were compared with the Log-Rank test. A two-sided *P* value of <0.05 was considered statistically significant. Analyses were performed using the SPSS software, version 18.0 (IBM Corporation, Armonk, NY, USA) and STATA 13 (STATA Inc., USA).

## Results

Baseline characteristics of the study group (n = 133) are shown in Table 1. Mean age was 56.9 ± 11.8 years (range 18–75 years) and 85 patients (63.9%) were male. PAF, PeAF and LSPeAF were diagnosed before ablation in 92, 32 and 9 patients, respectively. Hypertension was present in 51.1% of patients, diabetes mellitus in 9.8%, previous stroke or transient ischemic attack in 7.5%, ischemic heart disease in 5.3% and chronic obstructive pulmonary disease in 3.0% of patients. Before ablation, mean left ventricular (LV) ejection fraction (EF) was 60.5 ± 8.1% and mean LA diameter was 40.2 ± 5.2 mm. Bundle branch block (BBB) was recorded in 5 (4.4%) patients: left BBB in 3 and right BBB in 2 patients.

There were no statistically significant differences in patient demographics, comorbidities, AF characteristics or ablation lesion sets between the present study group and excluded patients, as shown in Supplementary Table S1.

### Early recurrence of AF (ERAF)

During the 3-month “blanking period” after ablation, an ERAF was documented in 25 (18.8%) patients, including PAF in 10 patients, paroxysmal AT in 8, paroxysmal typical AFL in 2, PeAF in 1 and persistent AT in 4 patients.

### Very late recurrence of AF (VLRAF)

**Figure 2:**
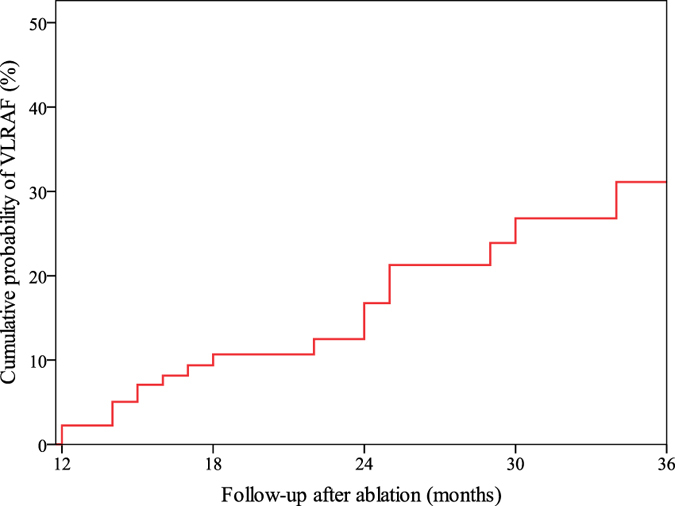
Progressive increase of VLRAF rate during follow-up after ablation procedure. VLRAF = very late recurrence of atrial fibrillation.

During the follow-up of 13.0 to 48.0 (mean 29.1 ± 10.1) months after the last procedure, VLRAF was documented in 20 (15.0%) patients, mostly by symptom triggered ECG (n = 15) and scheduled Holter-monitoring (n = 3), and only in two patients by additional follow-up examinations (event-recorder, n = 2). The overall VLRAF incidence rate of 16.3% per 100 patient-years at risk was determined. Figure 2 shows a progressive increase in VLRAF cumulative rate over time, from 16.8% (95%CI: 8.0–25.6%) at 2 years to 31.1% (95%CI: 17.0–45.2%) at 3 years after the procedure. VLRAF was clinically manifested as PAF in 7 patients, paroxysmal AT in 2, PeAF in 5 and persistent AT in 6 patients. The mean time from ablation to VLRAF was 20.7 ± 8.3 (13.0–42.0) months.

Patients with VLRAF more often were males and more often had a NPAF, BBB, the LA diameter of ≥47 mm, LA CFAE ablation, LA linear ablation, and ERAF (all p < 0.05) compared to patients without VLRAF (Table 1).

### The MB-LATER score

The association of baseline and follow-up parameters with VLRAF on univariate Cox proportional hazards regression analysis is shown in Table 1. Among the parameters that on univariate analysis were significantly associated with VLRAF during follow-up, we selected only clinical variables (i.e., Male gender, p = 0.031; BBB, p = 0.004; LA dimension ≥47 mm, p = 0.006; Type of AF, p = 0.001 for NPAF; and ER-AF, p = 0.017), which were used to formulate the MB-LATER (“May-Be-LATER”) score. Each component of the score was assigned 1 point, except for the type of AF which was assigned 0, 1 and 2 points for PAF, PeAF and LSPeAF, respectively (Table 2). Thus, the MB-LATER score could range from 0 to 6 points.

**Figure 3:**
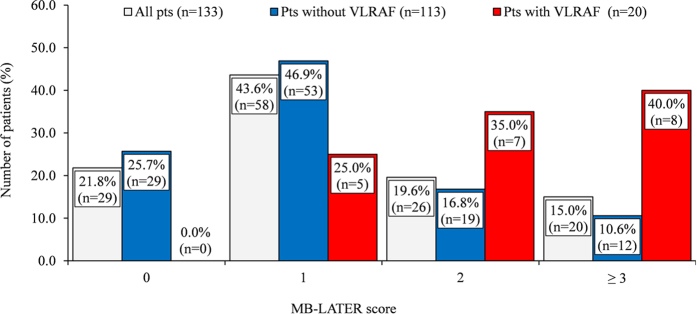
Distribution of MB-LATER score in the entire cohort of study group patients, in patients without VLRAF and in those with VLRAF. VLRAF = very late recurrence of atrial fibrillation.

The mean MB-LATER score was significantly higher in patients with VLRAF compared to those without VLRAF (2.4 ± 1.2 vs. 1.2 ± 1.1, p < 0.001). The distribution of patients with the VLRAF across the MB-LATER score strata was 0%, 25.0%, 35.0% and 40.0% with scores 0, 1, 2 and ≥3, respectively (Fig. 3).

**Figure 4:**
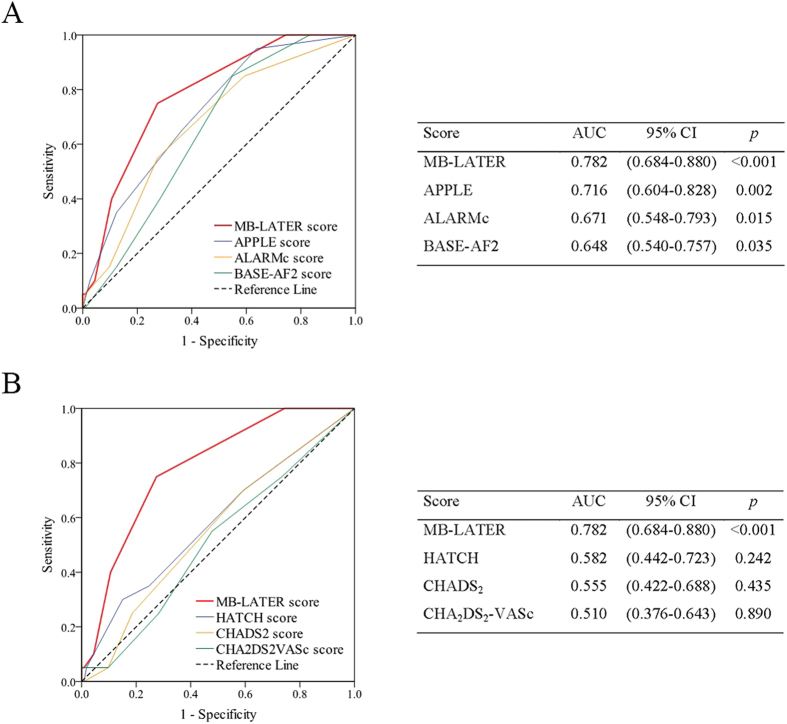
Comparison of various scoring systems for prediction of VLRAF. VLRAF = very late recurrence of atrial fibrillation; ROC = receiver operating characteristic; AUC = area under curve; CI = confidence interval.

**Figure 5:**
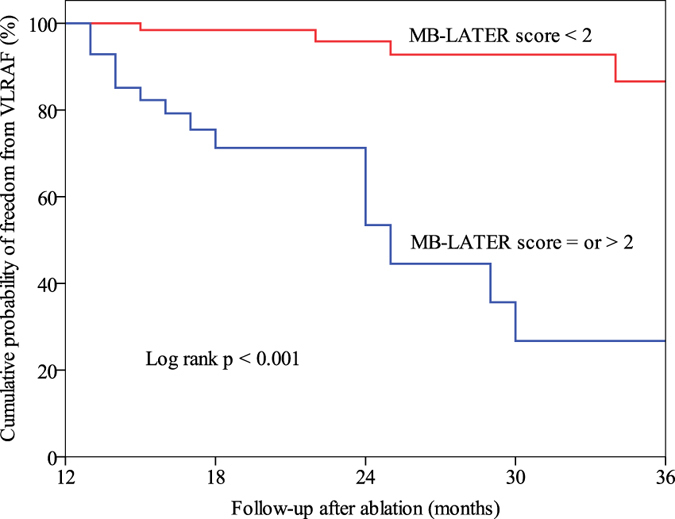
Kaplan-Meier curve showing VLRAF-free survival >1 year after ablation procedure according to cut-off value of MB-LATER score <2 or ≥2. VLRAF = very late recurrence of atrial fibrillation.

The MB-LATER score showed good predictive ability for VLRAF (AUC = 0.782; 95%CI, 0.684–0.880, p < 0.001), Fig. 4. On the cut-off analysis, a MB-LATER score of ≥2 had the best predictive value for VLRAF with 75.0% sensitivity, 72.6% specificity, a positive predictive value of 32.6% and negative predictive value of 94.3%. The AF-free survival rate >1 year after ablation was significantly higher in patients with a MB-LATER score of <2 than in those with a score of ≥2 (Log rank p < 0.001), Fig. 5.

**Figure 6:**
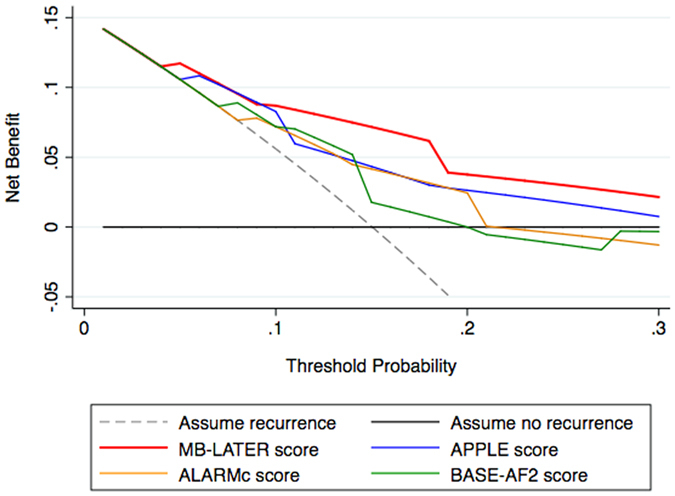
Decision curve for prediction of VLRAF. VLRAF, very late recurrence of atrial fibrillation.

In comparison to other scores proposed specifically for the assessment of the risk of AF recurrence post AF ablation (that is, the APPLE, ALARMc and BASE-AF2 score), or previously reported to predict recurrent AF post ablation (e.g., the CHADS_2_, CHA_2_DS_2_VASc or HATCH score), the MB-LATER score showed the best predictive ability for VLRAF in our cohort (Figs 4 and 6). The utilisation of the MB-LATER score significantly improved prediction of VLRAF in terms of IDI and NRI compared to other scores tested (Table 3). Relative to other scores, the use of the MB-LATER score would result in the NRI of 48.6–95.1% in predicting VLRAF (Table 3), and the decision-curve analysis (DCA) demonstrated better identification of patients who will have a VLRAF based on the predictions of the MB-LATER score in comparison with other scores (Fig. 6). Table 4 shows the univariate analysis of association between components of the specific scoring systems (MB-LATER, APPLE, ALARMc and BASE-AF2) and VLRAF occurrence.

### Sensitivity analysis

Predictive ability of the MB-LATER score in the sub-group of patients with first-ever AF ablation (n = 103) was broadly similar as in the main analysis (AUC = 0.769; 95%CI, 0.664–0.873, p < 0.001).

### The MB-LATER score validation (an exploratory analysis)

The validation cohort consisted of 39 patients, who were not included in the derivation cohort due to the short follow-up after the last procedure (i.e., a follow-up of ≤12 months). Until November 2016 these patients have qualified for the inclusion into a validation cohort, but only for the purpose of an exploratory analysis due to a small number of patients. The mean follow-up in the validation cohort was 14.6 ± 1.9 months (range 13.0–17.0 months). A total of three VLRAF cases were detected, manifested as PAF, PeAT and PAT (n = 1 each). The time from last procedure to VLRAF was 13.0, 13.0 and 15.0 months, respectively. There were no significant differences in baseline clinical characteristics between the derivation and validation cohort (supplementary Table S2). On exploratory analysis the MB-LATER score had a good predictive value for the occurrence of VLRAF after ablation (AUC = 0.833 [95%CI: 0.671–0.996], p = 0.058).

## Discussion

In the present study we identified common, readily available clinical variables (i.e., male gender, pre-procedural bundle branch block, LA anteroposterior diameter of ≥47 mm, non-paroxysmal pre-procedural AF and early AF recurrence post-ablation) which were significantly associated with increased risk of very late AF recurrence >12 months post ablation. Combining these simple clinical parameters into the MB-LATER score (**M**ale gender, **B**BB, **LA** dilatation ≥47 mm, **T**ype of AF [PAF, PeAF or LSPeAF] and **ER**AF), yielded a good predictive ability for VLRAF (AUC 0.782) in our study population. The use of the MB-LATER score was associated with better identification of patients who will have a VLRAF as compared to other scores such as the APPLE, ALARMc or BASE-AF2 risk score.

This is the first score specifically addressing patients free of AF recurrence at 1 year after ablation. In clinical practice, such patients are often considered as ‘cured’ and hence subjected to a less intensive further clinical follow-up beyond 1 year post ablation^1^,^2^,^3^,^8^,^10^. Importantly, the freedom from AF at 1 year post AF ablation may be tempting with respect to permanent discontinuation of oral anticoagulant therapy even in patients at increased risk of stroke, which could be prevented by reliable AF late recurrence risk assessment. Increased risk of late AF recurrence could also influence decision-making regarding the use of AADs and/or consideration of multiple re-do ablations^4^,^5^,^6^,^7^,^9^.

Three other scoring systems have been previously derived for the prediction of AF recurrence after ablation^10^,^11^,^12^. The ALARMEc score (NPAF, normalized LA area >10.25, eGFR <68 ml/min, metabolic syndrome and cardiomyopathy) had a good predictivity of AF recurrence during a 2-year follow-up after redo procedure (AUC: 0.657) and was superior to CHADS_2_ or CHA_2_DS_2_-VASc scores in this setting^11^. However, the score included a nonstandard definition of NPAF, renal failure, metabolic syndrome and LA enlargement^11^. The APPLE score (LA diameter ≥43 mm, PeAF, age >65 years, eGFR <60 mL/min/1.73 m^2^ and LVEF <50%) has also shown better predictive value for AF recurrence at 1 year following the first ablation (AUC: 0.634) than the CHADS_2_ or CHA_2_DS_2_-VASc scores (AUC: 0.538 and 0.542, respectively)^10^. The BASE-AF2 score ≥3 (body mass index >28 kg/m^2^, LA >40 mm, current smoking, ERAF, duration of AF history >6 years and NPAF) accurately predicted AF recurrence over a 20-month follow-up after cryo-balloon PV isolation (AUC: 0.94)^12^. However, these scores addressed *any* AF recurrence post ablation (and not specifically VLRAF in patients who were AF-free during the first post-procedural year), the score calculation required blood biomarkers values or other parameters which are not regularly obtained in routine clinical practice, and the follow-up in these studies was limited^10^,^11^,^12^.

Some of the MB-LATER score components, such as AF clinical type, LA dilatation and ERAF have been already recognized as independent predictors of AF recurrence following ablation and were incorporated in other scoring systems^7^,^10^,^11^,^12^,^17^,^18^. In our study, BBB was significantly associated with VLRAF. It has been recognized that QRS duration is a specific indicator of LV systolic dysfunction^19^. HF and male gender are well known risk factors for AF development^1^,^2^. Although APPLE, ALARMc and BASE-AF2 scores worked well in our study of younger patients with lone AF or a minimal cardiovascular disease, the MB-LATER score showed better predictive accuracy for VLRAF than other scores. In our study the procedures with longer fluoroscopy and ablation times were associated with a higher rate of VLRAF. Longer procedure times may reflect a more complex anatomy and substrate, but also they can be affected by technology and experience of the operator. Recently, it has been reported that the current trend towards the use of “near zero” approach is adopted in different electrophysiological procedures, in order to protect the patients and staff from the harmful effects of ionizing radiation^20^,^21^.

The CHADS_2_ and CHA_2_DS_2_-VASc scores originally proposed for the AF-related thromboembolic risk assessment have also been studied for the prediction of various clinical outcomes after AF ablation, including death, hospitalization for HF and short-term AF recurrence^22^,^23^. In addition, both scores have been shown to predict VLRAF after ablation in a study with a 5-year follow-up period of “older and sicker” AF patients, with a mean age of 65.7 years and high prevalence of certain comorbidities, which are constituents of these scores^22^. The components of CHADS_2_ and CHA_2_DS_2_-VASc scores are ‘universal’ cardiovascular risk factors non-specific for AF. Thus, the predictive value of these scores might be attenuated in younger patients with lone AF or minimal structural heart disease, who are actually the best candidates for PV isolation^1^,^2^. The HATCH score, originally established to predict the progression of PAF to PeAF, has shown limited clinical value for the prediction of arrhythmia recurrence following AF ablation^24^. Overall, in our study, the MB-LATER score had better predictive ability than the CHADS_2_, CHA_2_DS_2_VASc, HATCH, APPLE, ALARMc and BASE-AF2 scores. A MB-LATER score cut-off point of ≥2 had the highest predictive value for VLRAF.

### Incidence, mechanisms and risk factors for VLRAF

The annual incidence of VLRAF in patients who were AF-free at 1 year after ablation was 16.3% in our study, and the VLRAF cumulative incidence rate increased slowly, but continuously, reaching 31.1% at 3 years post procedure.

There are controversies about the durability of the AF ablation effects^2^,^4^,^5^,^6^,^7^,^8^,^9^. The AF recurrence mostly occurs in the first year after CA^1^,^2^, while in patients free of AF for ≥1 year after ablation variable rates of VLRAF have been reported, ranging between 6.7% and 8.9% during follow-up of 4.6–4.8 years^3^,^8^ or up to 41.2% of patients over a longer follow-up^4^,^6^. These findings indicate a progressive increase in the AF recurrence rate during long-term post-procedural follow-up with an average annual incidence of 7–10%^4^,^5^,^6^,^7^,^8^,^9^, in line with our results.

It has been suggested that PV reconnection is a primary cause of the AF recurrence within the first year after ablation^1^,^2^,^3^, while additional mechanisms other than simple PV reconnection could be responsible for at least some of VLRAF cases^4^,^5^,^8^,^9^,^25^,^26^. Indeed, a significantly lower prevalence of AF foci in the LA and PVs and higher prevalence of AF foci in the right atrium was found on EP study in the VLRAF patients compared to patients with an earlier AF recurrence^26^. At redo procedure, non-PV foci of AF were more often ablated in VLRAF patients than in those with LRAF (54% vs. 30%)^25^. Moreover, AF/AT unrelated to the PVs was diagnosed in 64.9% of patients with VLRAF^9^.

Numerous clinical variables were identified as independent predictors of VLRAF: non-paroxysmal AF, aging, LV systolic and diastolic dysfunction, high preoperative C-reactive protein level, LA enlargement, higher CHADS_2_ score, the presence of non-PV triggers, obesity, hypertension, hyperlipidaemia and ERAF^4^,^5^,^7^,^17^,^18^,^25^,^26^,^27^. It’s possible that these risk factors continue to affect the process of atrial remodelling despite initially “successful” ablation, leading to the substrate and triggers evolution over years, and the „very late“ reappearance of AF.

### Clinical implications

Given the high arrhythmia recurrence rate after ablation, significant procedural costs, long radiation exposure time and potentially serious complications that may accompany an AF ablation procedure, better selection of patients using MB-LATER score may be of clinical significance^1^,^2^. In addition, anticipation of delayed AF recurrence in patients with higher MB-LATER score could influence the post-procedural clinical decision to continue anticoagulation and AAD therapy and the methods and intensity of rhythm monitoring >1 year after successful CA. Presently, there are no clear strategies or recommendations for further rhythm monitoring in patients free from AF after the first post-procedural year. As per the Task Force on Catheter and Surgical Ablation of AF Consensus, “every patient should be seen in follow-up at a minimum of three months following the ablation procedure, and then every six months for at least two years”^1^. It has been recently reported that whilst the CHADS_2_, R_2_CHADS_2_ and CHA_2_DS_2_-VASc scores were independent predictors of thromboembolic events during post AF ablation follow-up, AF recurrence conferred only a non-significant trend for increased thromboembolic risk^28^. Hence, it is suggested to continue long-term oral anticoagulation therapy in all patients at high thromboembolic risk, i.e. CHA_2_DS_2_-VASc ≥2, regardless of the AF ablation outcome^29^. However, it has been shown recently that all thromboembolic events following AF ablation occur in patients who experienced arrhythmia recurrence (4% among patients with AF recurrence vs. 0% among those who were arrhythmia-free post procedure, p < 0.001)^29^. These findings emphasize the importance of an early detection of AF recurrence after ablation^1^,^3^,^4^,^5^,^6^,^7^,^8^,^9^,^10^,^11^,^12^,^17^,^18^,^28^,^29^. Therefore, identification of patients at high risk for the delayed AF re-occurrence could determine the strategy for long-term rhythm monitoring of patients for whom it was believed to have been “cured” based on the freedom from arrhythmia at 1 year post ablation.

### Study limitations

This was a retrospective single-centre study and number of patients was limited. Classification of AF recurrences to the LRAF (3–12 months) and the VLRAF (>1 year) was arbitrary, but in concordance with current reports^1^,^2^,^3^,^4^,^5^,^6^,^7^,^8^,^9^,^17^,^18^,^20^,^25^,^26^,^27^. An intermittent rhythm monitoring after ablation by only 24-hour Holter-recording may underestimate the AF recurrence rate, especially in asymptomatic cases, and undetected AF recurrence within the first postoperative year may lead to its misinterpretation as VLRAF in some patients^1^. However, all our patients underwent a pre-scheduled regular monitoring and clinical follow-up visits. The MB-LATER score was derived retrospectively from a single-centre cohort, but its validation in several other cohorts of patients undergoing AF ablation in different countries is underway. In addition, a prospective validation of the score is ongoing in our centre. Until we achieve the relevant cohort size, we herein reported only an interim, exploratory score validation analysis, suggesting a good performance of the MB-LATER score.

## Conclusions

In our study, a progressive increase in cumulative AF recurrence rate was documented during further follow-up of patients who were AF-free at 1 year post AF ablation, and the MB-LATER score provided a simple, readily available VLRAF risk assessment and performed better than other scores. Validation of the MB-LATER score in other cohorts is underway.

## Additional Information

**How to cite this article**: Mujović, N. *et al*. Prediction of very late arrhythmia recurrence after radiofrequency catheter ablation of atrial fibrillation: The MB-LATER clinical score. *Sci. Rep.* **7**, 40828; doi: 10.1038/srep40828 (2017).

**Publisher's note:** Springer Nature remains neutral with regard to jurisdictional claims in published maps and institutional affiliations.

## Supplementary Material

Supplementary Dataset 1

## Figures and Tables

**Table 1:** Baseline characteristics of the study cohort.

	All (n = 133)	Patients with VLRAF (n = 20)	Patients without VLRAF (n = 113)	Univariate analysis HR (95% CI)	p-value
Age (y)	56.9 ± 11.8	56.2 ± 15.4	57.0 ± 11.1	1.00 (0.96–1.04)	0.888
Males	85 (63.9%)	16 (80.0%)	69 (61.1%)	3.37 (1.12–10.12)	0.031
BMI (kg/m^2^)	27.5 ± 4.1	27.6 ± 2.6	27.5 ± 4.3	0.99 (0.91–1.09)	0.912
AF history (y)	7.2 ± 6.9	6.1 ± 5.6	7.5 ± 7.2	0.97 (0.90–1.04)	0.423
NPAF	41 (30.8%)	12 (60.0%)	29 (25.7%)	6.63 (2.55–17.24)	0.001
EHRA score	2.6 ± 0.7	2.4 ± 1.0	2.6 ± 0.7	0.63 (0.34–1.17)	0.140
PR (ms)	172.6 ± 31.9	180.7 ± 42.4	171.3 ± 30.0	0.99 (0.96–1.02)	0.347
QRS (ms)	102.1 ± 11.7	106.9 ± 16.7	101.3 ± 10.5	1.02 (0.99–1.05)	0.128
BBB	5 (4.4%)	3 (15.0%)	2 (1.8%)	6.08 (1.75–21.06)	0.004
LV EDD (mm)	52.0 ± 4.4	53.3 ± 4.0	51.8 ± 4.5	1.11 (1.00–1.23)	0.055
LV EF (%)	60.5 ± 8.1	57.6 ± 8.4	61.0 ± 8.0	0.96 (0.92–1.00)	0.077
LV EF < 50%	15 (11.3%)	3 (15.0%)	12 (10.6%)	2.08 (0.60–7.20)	0.248
LAD (mm)	40.2 ± 5.2	42.2 ± 5.0	39.9 ± 5.2	1.09 (0.99–1.20)	0.069
LAD ≥ 47 mm	15 (11.3%)	5 (25.0%)	10 (8.8%)	4.27 (1.50–12.15)	0.006
CHF	18 (13.5%)	5 (25.0%)	13 (11.5%)	2.12 (0.76–5.90)	0.149
IHD	7 (5.3%)	2 (10.0%)	5 (4.4%)	2.32 (0.53–10.12)	0.262
Hypertension	68 (51.1%)	13 (65.0%)	55 (48.7%)	1.85 (0.76–4.49)	0.176
DM	13 (9.8%)	2 (10.0%)	11 (9.7%)	1.20 (0.28–5.20)	0.809
COPD	4 (3.0%)	1 (0.5%)	3 (2.7%)	0.83 (0.11–6.45)	0.858
TIA/CVA	10 (7.5%)	0 (0.0%)	10 (8.8%)	0.04 (0.00–125.69)	0.442
Previous hyper-thyroidism	12 (9.0%)	1 (5.0%)	11 (9.7%)	0.72 (0.10–5.46)	0.753
CHA_2_DS_2_VASc score	1.7 ± 1.4	1.8 ± 1.5	1.7 ± 1.4	1.08 (0.77–1.51)	0.661
CHADS_2_ score	0.9 ± 1.0	1.0 ± 0.9	0.9 ± 1.0	1.21 (0.76–1.94)	0.428
HATCH score	1.1 ± 1.2	1.4 ± 1.3	1.0 ± 1.1	1.38 (0.94–2.04)	0.103
Failed AADs	2.7 ± 1.2	2.8 ± 0.7	2.7 ± 1.3	0.95 (0.66–1.35)	0.767
Amiodarone	89 (66.9%)	18 (90.0%)	71 (62.8%)	3.75 (0.86–16.25)	0.078
PV isolation	132 (99.2%)	20 (100.0%)	112 (99.1%)	0.54 (0.07–4.06)	0.552
SVC isolation	22 (16.5%)	1 (5.0%)	21 (18.6%)	0.31 (0.04–2.36)	0.258
LA CFAE abl.	28 (21.1%)	8 (40.0%)	20 (17.7%)	3.54 (1.42–8.84)	0.007
LA linear abl.	50 (37.6%)	12 (60.0%)	38 (33.6%)	4.02 (1.60–10.08)	0.003
CTI ablation	59 (44.4%)	10 (50.0%)	49 (43.4%)	1.41 (0.58–3.42)	0.452
X-ray (min)	37.2 ± 14.1	47.8 ± 12.2	35.2 ± 13.6	1.04 (1.00–1.07)	0.025
RF time (min)	76.1 ± 32.6	96.3 ± 24.6	72.5 ± 32.6	1.02 (1.00–1.03)	0.031
Redo procedure	30 (22.6%)	1 (5.0%)	29 (25.7%)	0.16 (0.02–1.19)	0.073
eGFR (ml/min)	81.8 ± 20.4	80.6 ± 21.1	81.9 ± 20.4	1.00 (0.98–1.02)	0.776
CRP (mg/l)	19.3 ± 28.6	29.7 ± 33.6	18.1 ± 27.9	1.00 (0.99–1.02)	0.590
Tn-T (μg/l)	5.2 ± 4.2	5.6 ± 5.4	5.1 ± 4.1	1.09 (0.95–1.24)	0.217
ERAF	25 (18.8%)	8 (40.0%)	17 (15.0%)	3.00 (1.22–7.37)	0.017
Beta-blockers	74 (55.6%)	14 (70.0%)	60 (53.1%)	2.14 (0.81–5.64)	0.123
ACEi /ARBs	53 (39.9%)	10 (50.0%)	43 (38.1%)	1.20 (0.50–2.93)	0.682
Statins	37 (27.8%)	6 (30.0%)	31 (27.4%)	0.98 (0.37–2.56)	0.964

Data are presented as mean ± standard deviation and percentages.

VLRAF = very late recurrence of atrial fibrillation; HR = hazard ratio; CI = confidence interval; BMI = body mass index; AF = atrial fibrillation; NPAF = non-paroxysmal AF; EHRA = European Heart Rhythm Association; BBB = bundle branch block; LV = left ventricle; EDD = enddiastolic dimension; EF = ejection fraction; LAD = left atrial diameter; CHF = congestive heart failure; IHD = ischemic heart disease; DM = diabetes mellitus; COPD = chronic obstructive pulmonary disease; TIA = transient ischemic attack; CVA = cerebrovascular accident; AAD = antiarrhythmic drug; PV = pulmonary vein; SVC = superior vena cava; LA = left atrium; CFAE = complex fractionated atrial electrogram; CTI = cavotricuspid isthmus; RF = radiofrequency; eGFR = estimated glomerular filtration rate; CRP = C-reactive protein; Tn-T = troponin-T; ERAF = early recurrence of AF; ACEi = angiotensin converting enzyme; ARB = angiotensin receptor blockers.

**Table 2:** The MB-LATER score.

**M**	Male gender	1
**B**	Bundle branch block	1
**LA**	Left Atrium ≥ 47 mm	1
**T**	Type (of AF)	0 = paroxysmal AF
		1 = persistent AF
		2 = long-standing persistent AF
**ER**	Early Recurrence (of AF)	1
**Maximum points**		**6**

AF = atrial fibrillation.

**Table 3:** Measures of predictive accuracy and improvement using MB-LATER score in prognostication of VLRAF.

MB-LATER score vs.	IDI	P-value	NRI	P-value
APPLE score	0.052	0.06	0.533	0.03
ALARMc score	0.080	0.006	0.486	0.05
BASE-AF2 score	0.105	0.005	0.715	0.003
HATCH score	0.119	0.002	0.951	<0.001
CHADS_2_ score	0.123	0.003	0.951	<0.001
CHA_2_DS_2_-VASc score	0.127	0.003	0.951	<0.001

IDI = Integrated Discrimination Improvement; NRI = Net Reclassification Improvement; VLRAF = very late recurrence of atrial fibrillation.

**Table 4:** Components of the scoring systems used for prediction of AF recurrence after ablation.

Score (points)	All (n = 133)	Patients with VLRAF (n = 20)	Patients without VLRAF (n = 113)	Univariate analysis		
				HR	(95% CI)	p-value
**MB-LATER** (0–6)	1.4 ± 1.2	2.4 ± 1.2	1.2 ± 1.1	2.49	(1.75–3.57)	<0.001
Male gender	85 (63.9%)	16 (80.0%)	69 (61.1%)	3.37	(1.12–10.12)	0.031
BBB	5 (4.4%)	3 (15.0%)	2 (1.8%)	6.08	(1.75–21.06)	0.004
LAD ≥ 47 mm	15 (11.3%)	5 (25.0%)	10 (8.8%)	4.27	(1.50–12.15)	0.006
Type of AF (NPAF)	41 (30.8%)	12 (60.0%)	29 (25.7%)	6.63	(2.55–17.24)	0.001
ERAF	25 (18.8%)	8 (40.0%)	17 (15.0%)	3.00	(1.22–7.37)	0.017
**APPLE**^10^ (0–5)	1.3 ± 1.2	2.1 ± 1.1	1.2 ± 1.1	1.95	(1.33–2.86)	0.001
Age > 65 years	36 (27.1%)	8 (40.0%)	28 (24.8%)	1.95	(0.80–4.77)	0.144
PeAF*	41 (30.8%)	12 (60.0%)	29 (25.7%)	6.63	(2.55–17.24)	<0.001
LAD ≥ 43 mm	48 (36.1%)	9 (45.0%)	39 (34.5%)	1.35	(0.56–3.27)	0.505
LVEF < 50%	10 (7.5%)	3 (15.0%)	7 (6.2%)	2.08	(0.60–7.20)	0.248
eGFR < 60 ml/min/1.73 m2	36 (27.1%)	9 (45.0%)	27 (23.9%)	2.23	(0.92–5.39)	0.076
**ALARMc**^11^ (0–5)	1.1 ± 1.0	1.6 ± 1.1	1.0 ± 1.0	2.07	(1.29–3.31)	0.003
NPAF†	41 (30.8%)	12 (60.0%)	29 (25.7%)	6.63	(2.55–17.24)	<0.001
LAD index (mm/m^2^) > 23‡	14 (10.5%)	5 (25.0%)	9 (8.0%)	2.88	(1.04–7.99)	0.043
eGFR < 68 ml/min	30 (22.6%)	6 (30.0%)	24 (21.2%)	1.35	(0.52–3.55)	0.537
MeS	47 (35.3%)	6 (30.0%)	41 (36.3%)	0.81	(0.31–2.11)	0.661
Cardiomyopathy	10 (7.5%)	3 (15.0%)	7 (6.2%)	2.08	(0.60–7.20)	0.248
**BASE-AF2**^12^ (0–6)	1.9 ± 1.3	2.4 ± 0.9	1.8 ± 1.3	1.60	(1.10–2.34)	0.014
BMI > 28 kg/m^2^	48 (36.1%)	7 (35.0%)	41 (36.3%)	0.92	(0.36–2.34)	0.855
LAD > 40 mm	65 (48.9%)	12 (60.0%)	53 (46.9%)	1.63	(0.67–4.00)	0.284
Cigarette smoking	20 (15.0%)	2 (10.0%)	18 (15.9%)	0.63	(0.15–2.73)	0.536
ERAF	25 (18.8%)	8 (40.0%)	17 (15.0%)	3.00	(1.22–7.37)	0.017
AF history > 6 years	52 (39.1%)	7 (35.0%)	45 (39.8%)	0.94	(0.37–2.39)	0.859
NPAF	41 (30.8%)	12 (60.0%)	29 (25.7%)	6.63	(2.55–17.24)	<0.001

Data are presented as numbers with percentages.

VLRAF = very late recurrence of atrial fibrillation (>12 months after ablation); HR = hazard ratio; CI = confidence interval; BBB = bundle branch block; LAD = left atrial diameter; AF = atrial fibrillation; NPAF = non-paroxysmal AF; ERAF = early recurrence of AF (within the 3-month “blanking period”); PeAF = persistent AF; LVEF = left ventricular ejection fraction; eGFR = estimated glomerular filtration rate; MeS = metabolic syndrome; BMI = body mass index.

^*^In the study evaluating APPLE score^10^, PeAF included patients with persistent and longstanding persistent AF.

^†^In the study evaluating ALARM score^11^, NPAF was defined as persistent, long-standing persistent and paroxysmal AF in patients with >500 h in symptomatic AF within 3 months prior to admission, and left atrial enlargement was defined as normalized left atrial area > 10.25.

^‡^In our analysis, LAD index (mm/m^2^) was used as an approximation for normalized left atrial area, because of the retrospective study design only data on antero-posterior LAD were available. We defined cardiomyopathy as LVEF <50%.
