# Supplementary material for: Prediction of very late arrhythmia recurrence after radiofrequency catheter ablation of atrial fibrillation: The MB-LATER clinical score
Source: Sci Rep. 2017 Jan 20;7:40828. doi: 10.1038/srep40828 (PMC5247745; doi:10.1038/srep40828)
Supplement: Supplementary Dataset 1 [file srep40828-s1.doc]

**APPENDIX (Supplement)**

**to**

**Clinical research**

**Prediction of very late arrhythmia recurrence after radiofrequency catheter ablation of atrial fibrillation: The MB-LATER clinical score**

*Nebojša Mujović^1,2^, Milan Marinković^1^, Nebojša Marković^1^, Alena Shantsila^3^, Gregory Y.H. Lip^2,3^, *Tatjana S. Potpara^1, 2^.

^1^ Cardiology Clinic, Clinical Center of Serbia, Višegradska 26, Belgrade, Serbia;

^2^ School of Medicine, University of Belgrade, Dr Subotića 8, Belgrade, Serbia;

^3^ University of Birmingham Institute of Cardiovascular Science, City Hospital, Birmingham, United Kingdom.

**Table S1.** Baseline characteristics of all patients.

|  | All patients  (n=212) | Study group  (n=133) | Excluded patients  (n=79) | p-value |
| --- | --- | --- | --- | --- |
| Age (years) | 57.4±11.3 | 56.9±11.8 | 58.1±10.5 | 0.541 |
| Male gender | 137 (64.6%) | 85 (63.9%) | 52 (65.8%) | 0.778 |
| BMI (kg/m^2^) | 27.3±4.1 | 27.5±4.1 | 27.2±4.1 | 0.607 |
| Years since first-diagnosed AF | 7.3±6.8 | 7.2±6.9 | 7.5±6.4 | 0.520 |
| Non-paroxysmal AF | 73 (34.4%) | 41 (30.8%) | 32 (40.5%) | 0.152 |
| EHRA symptom score | 2.5±0.7 | 2.6±0.7 | 2.5±0.7 | 0.182 |
| PR-interval (ms) | 172.1±29.4 | 172.6±31.9 | 171.6±33.7 | 0.829 |
| QRS-complex duration (ms) | 101.9±10.4 | 102.1±11.7 | 100.4±12.3 | 0.711 |
| Bundle branch block | 10 (4.7%) | 5 (4.4%) | 5 (6.3%) | 0.393 |
| LV EDD (mm) | 52.1±4.2 | 52.0±4.4 | 52.3±3.7 | 0.521 |
| LV EF (%) | 60.3±8.4 | 60.5±8.1 | 60.1±8.9 | 0.698 |
| LV EF < 50 % | 14 (6.6%) | 10 (7.5%) | 4 (5.1%) | 0.577 |
| LA diameter (mm) | 40.8±5.3 | 40.2±5.2 | 41.8±5.4 | 0.085 |
| LA diameter ≥ 47 mm | 27 (12.7%) | 15 (11.3%) | 12 (15.2%) | 0.409 |
| Congestive heart failure | 28 (13.2%) | 18 (13.5%) | 10 (12.7%) | 0.856 |
| Ischemic heart disease | 13 (6.1%) | 7 (5.3%) | 6 (7.6%) | 0.494 |
| Hypertension | 110 (51.9%) | 68 (51.1%) | 42 (53.2%) | 0.774 |
| Diabetes mellitus | 21 (9.9%) | 13 (9.8%) | 8 (10.1%) | 0.934 |
| COPD | 4 (1.9%) | 4 (3.0%) | 0 (0.0%) | 0.299 |
| Previous TIA/CVA | 16 (7.6%) | 10 (7.5%) | 6 (7.6%) | 0.984 |
| Previous hyperthyroidism | 18 (8.5%) | 12 (9.0%) | 6 (7.6%) | 0.702 |
| CHA_2_DS_2_-VASc score | 1.7±1.4 | 1.7±1.4 | 1.8±1.4 | 0.667 |
| HATCH score | 1.1±1.1 | 1.1±1.2 | 1.1±1.0 | 0.703 |
| Failed AADs | 2.7±1.2 | 2.7±1.2 | 2.7±1.1 | 0.892 |
| Amiodarone before proc. | 142 (67.0%) | 89 (66.9%) | 53 (67.1%) | 0.976 |
| PV isolation | 208 (98.1%) | 132 (99.2%) | 76 (96.2%) | 0.147 |
| SVC isolation | 30 (14.2%) | 22 (16.5%) | 8 (10.1%) | 0.275 |
| LA CFAE ablation | 48 (22.6%) | 28 (21.1%) | 20 (25.3%) | 0.584 |
| LA linear ablation | 84 (39.6%) | 50 (37.6%) | 34 (43.0%) | 0.433 |
| CT isthmus ablation | 91 (42.9%) | 59 (44.4%) | 32 (40.5%) | 0.164 |
| Redo procedure(s) | 52 (25.9%) | 30 (22.6%) | 25 (31.6%) | 0.194 |
| Fluoroscopy (min) | 36.5±24.9 | 37.2±14.1 | 35.3±36.9 | 0.005 |
| RF time (min) | 69.5±33.7 | 76.1±32.6 | 57.5±32.5 | <0.001 |
| eGFR (mL/min) | 82.7±21.8 | 81.8±20.4 | 84.2±24.0 | 0.942 |
| eGFR <60 mL/min | 25 (11.8%) | 17 (12.8%) | 8 (10.1%) | 0.562 |
| CRP after ablation (mg/L) | 17.2±23.8 | 19.3±28.6 | 14.2±14.5 | 0.419 |
| Tn-T after ablation (μg/L) | 5.6±5.0 | 5.2±4.2 | 6.4±6.4 | 0.472 |
| Early AF recurrence | 46 (21.7%) | 25 (18.8%) | 21 (26.6%) | 0.184 |
| Beta-blockers after proc. | 133 (62.7%) | 74 (55.6%) | 59 (74.7%) | 0.006 |
| ACEi or ARBs after proc. | 89 (42.0%) | 53 (39.9%) | 36 (45.6%) | 0.415 |
| Statins after proc. | 57 (26.9%) | 37 (27.8%) | 20 (25.3%) | 0.691 |

Data are presented as mean±standard deviation or numbers (percentage).

BMI=body mass index; AF=atrial fibrillation; EHRA=European Heart Rhythm Association; LV=left ventricle; EDD=end-diastolic dimension; EF=ejection fraction; LA=left atrium; COPD=chronic obstructive pulmonary disease; TIA=transitory ischemic attack; CVA=cerebrovascular accident; AAD=antiarrhythmic drug; PV=pulmonary vein; SVC=superior vena cava; CFAE=complex fractionated atrial electrogram; CT=cavo-tricuspid; RF=radiofrequency; eGFR=estimated glomerular filtration rate; CRP=C-reactive protein; Tn-T=troponin; ACEi=angiotensin converting enzyme inhibitor; ARB=angiotensin receptor blocker.

**Table S2.** Baseline characteristics of the study group and validation cohort.

|  | Study group  (n=133) | Validation cohort  (n=39) | p-value |
| --- | --- | --- | --- |
| Age (years) | 56.9±11.8 | 56.9±10.9 | >0.999 |
| Male gender | 85 (63.9%) | 27 (69.2%) | 0.673 |
| BMI (kg/m^2^) | 27.5±4.1 | 27.9±4.6 | 0.323 |
| Years since first-diagnosed AF | 7.2±6.9 | 6.0±5.4 | 0.303 |
| Non-paroxysmal AF | 41 (30.8%) | 19 (48.7%) | 0.061 |
| EHRA symptom score | 2.6±0.7 | 2.4±0.7 | 0.119 |
| Bundle branch block | 5 (4.4%) | 1 (2.6%) | >0.999 |
| LV EDD (mm) | 52.0±4.4 | 52.2±4.3 | 0.417 |
| LV EF (%) | 60.5±8.1 | 58.5±7.7 | 0.187 |
| LV EF < 50 % | 10 (7.5%) | 4 (10.3%) | 0.524 |
| LA diameter (mm) | 40.2±5.2 | 40.6±5.8 | 0.338 |
| LA diameter ≥ 47 mm | 15 (11.3%) | 6 (15.4%) | 0.681 |
| Congestive heart failure | 18 (13.5%) | 4 (10.3%) | 0.787 |
| Ischemic heart disease | 7 (5.3%) | 2 (5.1%) | >0.999 |
| Hypertension | 68 (51.1%) | 21 (53.9%) | 0.907 |
| Diabetes mellitus | 13 (9.8%) | 5 (12.8%) | 0.803 |
| COPD | 4 (3.0%) | 0 (0.0%) | 0.575 |
| Previous TIA/CVA | 10 (7.5%) | 1 (2.6%) | 0.460 |
| Previous hyperthyroidism | 12 (9.0%) | 3 (7.7%) | >0.999 |
| CHA_2_DS_2_-VASc score | 1.7±1.4 | 1.7±1.5 | 0.992 |
| HATCH score | 1.1±1.2 | 1.1±1.2 | 0.992 |
| Failed AADs | 2.7±1.2 | 2.4±1.2 | 0.116 |
| Amiodarone before proc. | 89 (66.9%) | 22 (56.4%) | 0.310 |
| PV isolation | 132 (99.2%) | 39 (100.0%) | >0.999 |
| SVC isolation | 22 (16.5%) | 3 (7.7%) | 0.205 |
| LA CFAE ablation | 28 (21.1%) | 6 (15.4%) | 0.580 |
| LA linear ablation | 50 (37.6%) | 22 (56.4%) | 0.056 |
| CT isthmus ablation | 59 (44.4%) | 11 (28.2%) | 0.105 |
| Redo procedure(s) | 30 (22.6%) | 7 (18.0%) | 0.693 |
| Fluoroscopy (min) | 37.2±14.1 | 30.6±10.7 | 0.007 |
| RF time (min) | 76.1±32.6 | 64.0±23.4 | 0.047 |
| eGFR (mL/min) | 81.8±20.4 | 87.9±19.1 | 0.097 |
| CRP after ablation (mg/L) | 19.3±28.6 | 12.3±7.8 | 0.133 |
| Tn-T after ablation (μg/L) | 5.2±4.2 | 6.4±4.7 | 0.067 |
| Early AF recurrence | 25 (18.8%) | 7 (18.0%) | 0.905 |
| Beta-blockers after proc. | 74 (55.6%) | 21 (54.0%) | 0.988 |
| ACEi or ARBs after proc. | 53 (39.9%) | 18 (46.2%) | 0.604 |
| Statins after proc. | 37 (27.8%) | 8 (20.5%) | 0.480 |
| MB-LATER score | 1.4±1.2 | 1.6±1.1 | 0.177 |

Data are presented as mean±standard deviation or numbers (percentage).

BMI=body mass index; AF=atrial fibrillation; EHRA=European Heart Rhythm Association; LV=left ventricle; EDD=end-diastolic dimension; EF=ejection fraction; LA=left atrium; COPD=chronic obstructive pulmonary disease; TIA=transitory ischemic attack; CVA=cerebrovascular accident; AAD=antiarrhythmic drug; PV=pulmonary vein; SVC=superior vena cava; CFAE=complex fractionated atrial electrogram; CT=cavo-tricuspid; RF=radiofrequency; eGFR=estimated glomerular filtration rate; CRP=C-reactive protein; Tn-T=troponin; ACEi=angiotensin converting enzyme inhibitor; ARB=angiotensin receptor blocker.
